# Pharmacokinetics and pharmacodynamics of propofol in cancer patients undergoing major lung surgery

**DOI:** 10.1007/s10928-015-9404-6

**Published:** 2015-01-28

**Authors:** Krzysztof Przybyłowski, Joanna Tyczka, Damian Szczesny, Agnieszka Bienert, Paweł Wiczling, Katarzyna Kut, Emilia Plenzler, Roman Kaliszan, Edmund Grześkowiak

**Affiliations:** 1 Department of Clinical Pharmacy and Biopharmacy, Karol Marcinkowski University of Medical Sciences, Poznan, Poland; 2 Intensive Care Department, Pulmonary Diseases and Thoracic Surgery Center, Poznan, Poland; 3 Department of Biopharmaceutics and Pharmacokinetics, Medical University of Gdansk, Al. Gen. J. Hallera 107, 80-416 Gdańsk, Poland

**Keywords:** Propofol, PK/PD, AAI index, Cancer patients, Major lung surgery

## Abstract

Despite the growing number of cancer cases and cancer surgeries around the world, the pharmacokinetics (PK) and pharmacodynamics (PD) of anesthetics used in this population are poorly understood. Patients operated due to cancer are usually in severe state and often require chemotherapy. It might affect the PK/PD of drugs used in this population. Therefore, in this study we explored the PK/PD of propofol in cancer patients having a major lung surgery. 23 patients that underwent a propofol–fentanyl total intravenous anesthesia were included in the analysis. A large set of demographic, biochemical and hemodynamic parameters was collected for the purpose of covariate analysis. Nonlinear mixed effect modeling in NONMEM was used to analyze the collected data. A three-compartment model was sufficient to describe PK of propofol. The anesthetic effect (AAI index) was linked to the propofol effect site concentrations through a sigmoidal *E*_*max*_ model. A slightly higher value of clearance, a lower value of distribution clearance, and a decreased volume of peripheral compartment were observed in our patients, as compared with the literature values reported for healthy volunteers by Schnider et al. and by Eleveld et al. Despite these differences, both models led to a clinically insignificant bias of −8 and −1 % in concentration predictions, as reflected by the median performance error. The *C*_*e50*_ and propofol biophase concentration at the time of postoperative orientation were low and equaled 1.40 and 1.13 mg/L. The population PK/PD model was proposed for cancer patients undergoing a major lung surgery. The large body of studied covariates did not affect PK/PD of propofol significantly. The modification of propofol dosage in the group of patients under study is not necessary when TCI-guided administration of propofol by means of the Schnider model is used.

**Electronic supplementary material:**

The online version of this article (doi:10.1007/s10928-015-9404-6) contains supplementary material, which is available to authorized users.

## Introduction

Cancer remains a significant cause of morbidity and mortality around the world. In Europe lung cancer is the most common neoplasm and the leading cause of death due to oncologic diseases in men and the second most frequent in women. At an early stage of the disease the resection of the affected lobe or pneumonectomy is the treatment of choice. Propofol is widely used in all kinds of surgeries due to its short effect and rapid recovery. Additionally, it is recommended in thoracic anesthesia to prevent pollution of the operating theatre and to reduce hypoxemia during one-lung ventilation [1–3]. Propofol is a highly lipophilic drug, with a large volume of distribution and high hepatic extraction ratio. It is rapidly metabolized, mainly by the liver, by glucuronidation and oxidation [1]. In clinical practice target controlled infusion (TCI) devices are often used to administer propofol. Currently available pharmacokinetic protocols for propofol were developed on the basis of studies conducted on healthy individuals [4]. However, both the disposition and response to any drug may be altered in clinical conditions. Patients operated due to lung cancer are usually at advanced age, have experienced significant loss of weight or undergone neoadjuvant chemotherapy resulting in anemia, hypoalbuminemia and altered organ function. Ischemic heart disease leading to impaired cardiac contractility is also common [3].

Propofol pharmacokinetic and pharmacodynamic properties are subject to high inter-individual variability [5–10]. Numerous factors have been found to influence the PK/PD of propofol, e.g. blood parameters, body weight, the overall condition of a patient and co-administered drugs [5–10]. Taking this into account, it is important to identify as many factors influencing the PK and PD of propofol as possible to improve the safety of its use [11]. Also, the potential effect of anesthesia on long-term patient outcome is increasingly acknowledged [12–14].

Our goal was to propose a population model of the PK/PD of propofol in cancer patients with physical status ASA III, undergoing a lung surgery and to test the effect of various covariates on the PK/PD parameters of propofol. We also compared our data with the Schnider et al. [15] and Eleveld et al. [16] models. The Schnider model was developed on the basis of data from healthy volunteers and it uses total body weight, age, height and lean body mass as covariates. It is currently incorporated in commercial target-controlled infusion pumps for the administration of propofol. The Eleveld model has been the most comprehensive model published so far, as it integrates 21 propofol datasets from children, adults, elderly and obese individuals, both healthy volunteers and patients.

## Materials and methods

### Subjects and study design

The data for pharmacokinetic and pharmacodynamic analysis were obtained from twenty three ASA III patients scheduled for a major lung surgery due to lung cancer between December 2010 and September 2011. The study was performed after the approval of the Research Ethics Committee at the University of Medical Sciences (Poznań, Poland) and written informed consent. The exclusion criteria were as follows: excessive alcohol intake, drug abuse, mental retardation, psychiatric disturbance and subjective hearing impairment. Oral premedication with 7.5 mg midazolam and thoracic epidural analgesia were used in all cases. Before the induction of anesthesia a 20-gauge radial arterial catheter was inserted under local anesthesia to provide continuous hemodynamic monitoring and to collect blood samples. Thoracic epidural anesthesia was performed at level T5 with a 6 mL bolus volume containing 0.1 mg fentanyl and 20 mg bupivacaine, followed by 0.125 % bupivacaine infusion at 4–6 mL/h. Anesthesia was induced with fentanyl (3 µg/kg, Polfa, Warsaw, Poland) and propofol (2 mg/kg, Plofed, Polfa Warsaw, Poland) followed by the continuous infusion of propofol at a rate of 8 mg/kg/h. Rocuronium 0.6 mg/kg (Esmeron, Organon) was administered to facilitate endobronchial intubation. Blood samples were collected from the radial artery 1, 2, 3, 5, 10, 15, 20, 30, 40, 45, 50, 60, 75, 90 and 120 min after the beginning of infusion and 3, 5, 15, 30, 60 and 120 min after the termination of propofol infusion. Blood was collected into heparinized tubes and centrifuged immediately. Plasma was stored at 4 °C. The propofol concentration in the plasma was assayed within 8 weeks by means of a high-performance liquid chromatography fluorescence detector [17–19]. The analytical procedure was validated with the within-day and between-day variation coefficients, which were <10 %. The lower limit of quantification (LLOQ) was 0.01 mg/L.

The depth of anesthesia was measured with AEP/2 Monitor (Danmeter, Denmark, software version 1.6). The AAI (A-line ARX-Index), an index reflecting changes in middle-latency auditory evoked potentials (AEP) was selected as a pharmacodynamic response quantifying the effect of propofol on the central nervous system. The AEP/2 Monitor recorded the bioelectrical activity of the auditory cortex in response to auditory stimuli. The AAI index was scaled as previously suggested by Vereecke et al. [20]. A baseline AAI index was obtained during a 5-min period before anesthesia, when the patient was lying quietly, with eyes closed and breathing 100 % oxygen via a face mask. The infusion of propofol was adjusted to achieve AAI 15–25. Additional routine anesthesia monitoring included continuous ECG monitoring, end-tidal capnography and pulse-oximetry. Various clinical parameters, including systolic and diastolic blood pressure, heart rate and blood parameters were measured and recorded to assess their effects on the PK and PD parameters of propofol.

### PK/PD modeling methods

Population nonlinear mixed-effect modeling was performed using NONMEM software (Version 7.2.0; ICON Development Solutions, Ellicott City, MD, USA), and the gfortran compiler 9.0. NONMEM runs were executed using Wings for NONMEM (WFN720; http://wfn.sourceforge.net). The first-order conditional estimation with interaction (FOCEI) method was used. The minimum value of the NONMEM objective function (OFV), typical goodness-of-fit diagnostic plots, and evaluation of the precision of PK parameter and variability estimates were used to discriminate between various models during the model-building process. The shrinkage was evaluated for all model parameters to assess if and to what degree the individual parameters “shrink” toward the population values. In general the shrinkage of inter-individual parameters lower than about 20 % suggests that the data is highly informative about the individual-predicted parameters [21]. The NONMEM data processing and plots were done in Matlab Software (Version 7.13; The MathWorks, Natick, MA, USA).

### PK/PD model

A sequential PK/PD analysis was performed. At first plasma propofol concentrations were described by means of a three-compartment model using ADVAN6 subroutine:

1 $$V_{P} \frac{{dC_{P} }}{dt} = R_{0} - CLC_{P} - Q_{1} C_{P} + Q_{1} C_{T1} - Q_{2} C_{P} + Q_{2} C_{T2} \;\;\;\;\;\;C_{P} (0) = 0$$

2 $$V_{T1} \frac{{dC_{T1} }}{dt} = Q_{1} C_{P} - Q_{1} C_{T1} \;\quad C_{T1} (0) = 0$$

3 $$V_{T2} \frac{{dC_{T2} }}{dt} = Q_{2} C_{P} - Q_{2} C_{T2} \quad C_{T2} (0) = 0$$

where *C*_*P*_, *C*_*T1*_ and *C*_*T2*_ denotes concentrations of propofol in central and both peripheral compartments. The model was parameterized with volume and clearance terms. *V*_*P*_, *V*_*T1*_ and *V*_*T2*_ denote volumes of distribution of the respective compartments, *CL* denotes metabolic clearance of propofol and *Q*_*1*_ and *Q*_*2*_ denote the inter-compartmental clearances. *R*_*0*_ denotes the infusion rate and all extra boluses that were given to a patient.

In the second step of the model building process, PK parameters were fixed to individual estimates and used as a driving force for the pharmacodynamic model. The AAI index was described by the effect compartment linked with a sigmoidal *E*_*max*_ model:

4 $$AAI = AAI_{0} \left( {1 - \frac{{E_{\hbox{max} } C_{e}^{\gamma } }}{{{\text{Ce}}_{50}^{\gamma } + C_{e}^{\gamma } }}} \right)$$

In this equation, *AAI*_*0*_ denotes the baseline (pretreatment) value of AAI, *E*_*max*_ denotes the maximal effect fixed to 1, and *Ce*_*50*_ denotes the concentration of propofol which produces 50 % of maximal effect and *C*_*e*_ denotes the concentration of propofol in the effect compartment, which was described by:

5 $$\frac{{dC_{e} }}{dt} = k_{e0} \cdot C_{P} - k_{e0} \cdot C_{e}$$

where *k*_*e0*_ denoted the effect compartment distribution rate constant.

Inter-individual variability (IIV) for all PK parameters was modeled assuming log normal distribution:

6 $$P_{i} = \theta_{P} \exp (\eta_{P,i} )$$

where *P* is the individual parameter, *θ*_*P*_ is the typical value of this parameter in the population, and *η*_*P*_ is a random effect for that parameter with the mean 0 and variance *ω*_*P*_^2^. The observed concentration of propofol and AAI were defined by:

7 $$C_{P,obs} = C_{P} (1 + \varepsilon_{prop,C} )$$

8 $$AAI_{obs} = AAI(1 + \varepsilon_{prop,AAI} ) + \varepsilon_{add,AAI}$$

where *C*_*P*_, AAI are defined by basic structural model and *ε*_*prop,C*_, *ε*_*add,AAI*_ and *ε*_*prop,AAI*_ represent the proportional random error for PK measurements, and additive and proportional residual random errors for AAI index. It was assumed that *ε* is normally distributed with the mean of 0 and variances denoted by *σ* ^2^.

### Handling the AAI index measurements with upper limit

The highest possible signal obtained from AAI measurements equaled 60. It was a consequence of using a reduced upper scale that truncates all higher signals due to their large inter-patients variability and lack of information on loss of consciousness [20]. The Beal M3 method with the F-FLAG option was used to consider the truncated AAI index measurements [22].

### Covariates search

The effect of various covariates on PK and PD of propofol was tested it this study. The covariate search was performed by plotting individual estimates of the PK/PD parameters against time independent covariates to identify their influence. If a relationship was found, it was described by means of a linear regression or a power model. The categorical covariates (i.e. gender) were included into the model based on indicator variables. Similarly all the time-dependent covariates were tested using a linear regression or a power model. The time-dependent covariates were heart rate, systolic and diastolic blood pressure. The significance of potential covariates was systematically evaluated in a stepwise forward selection (ΔOFV < 3.84 points, p < 0.05) followed by backward elimination (ΔOFV < 6.63 points, p < 0.01).

### Bootstrap

Evaluation of model robustness was based on the non-parametric bootstrapping with 1,000 replicates. From the bootstrap empirical posterior distribution, 90 % confidence intervals (5th–95th percentile) were obtained for the parameters as described by Parke et al. [23].

### Visual predictive check

The model performance was assessed by means of visual predictive check (VPC). The VPC was calculated based on 1,000 datasets simulated with the final parameter estimates. The different dosing regimens and variable infusion length required the use of prediction corrected VPC (pcVPC). The pcVPC’s were created by correcting the observed and simulated values for the average population prediction in the time-bin divided by population predictions for each observed and simulated value [24]. In this study the 10th, 50th and 90th percentile were used to summarize the data and VPC prediction. The pcVPC allow to compare the confidence intervals obtained from prediction with the observed data over time. When the corresponding percentile from the observed data falls outside the 95 % confidence interval derived from predictions, it is an indication of a model misspecification. Since the PK/PD data deviated to some extent from nominal times a binning across time was done.

### Recovery from anesthesia

The model-predicted plasma concentrations, biophase concentrations, and AAI index of propofol were determined from the final model at the time of postoperative orientation (time of awakening). The patients were asked loudly for name and place every minute after the infusion was stopped without physical stimulation. The obtained values were further summarized as median and range. The calculation were done using Matlab Software (Version 7.13; The MathWorks, Natick, MA, USA). Further, we sought to investigate the association between the time of awakening and time that biophase concentrations remain above the Ce50, Ce20, end Ce10.

### Model assessment

The proposed model, the most often used compartment model of propofol in TCI devices published by Schnider et al. [15], and the model proposed by Eleveld et al. [16] were used to check their performance in predicting propofol concentrations for patients undergoing major lung surgery. The prediction error (PE) was calculated for each measurement as PE = 100 (measured − predicted)/predicted, and was summarized as median for each individual. The median prediction error (MDPE) and median absolute prediction error (MDAPE) were calculated according to the formulas:

9 $$\begin{gathered} MDPE = median\left( {PE_{1} ,PE_{2} , \ldots PE_{n} } \right) \hfill \\ MDAPE = median\left( {\left| {PE_{1} } \right|,\left| {PE_{2} } \right|, \ldots \left| {PE_{n} } \right|} \right) \hfill \\ \end{gathered}$$

where *n* denotes number of subjects. MDPE reflects the bias of the model, whereas MDAPE reflects the inaccuracy of the prediction. The MDPE in a range from −20 to 20 % an MDPE less than 30 % during TCI are typically treated as acceptable as originally proposed by Glass et al. [25]. The similar criteria for model comparison were used in the work of Masui et al. [26].

## Results

**Fig. 1:**
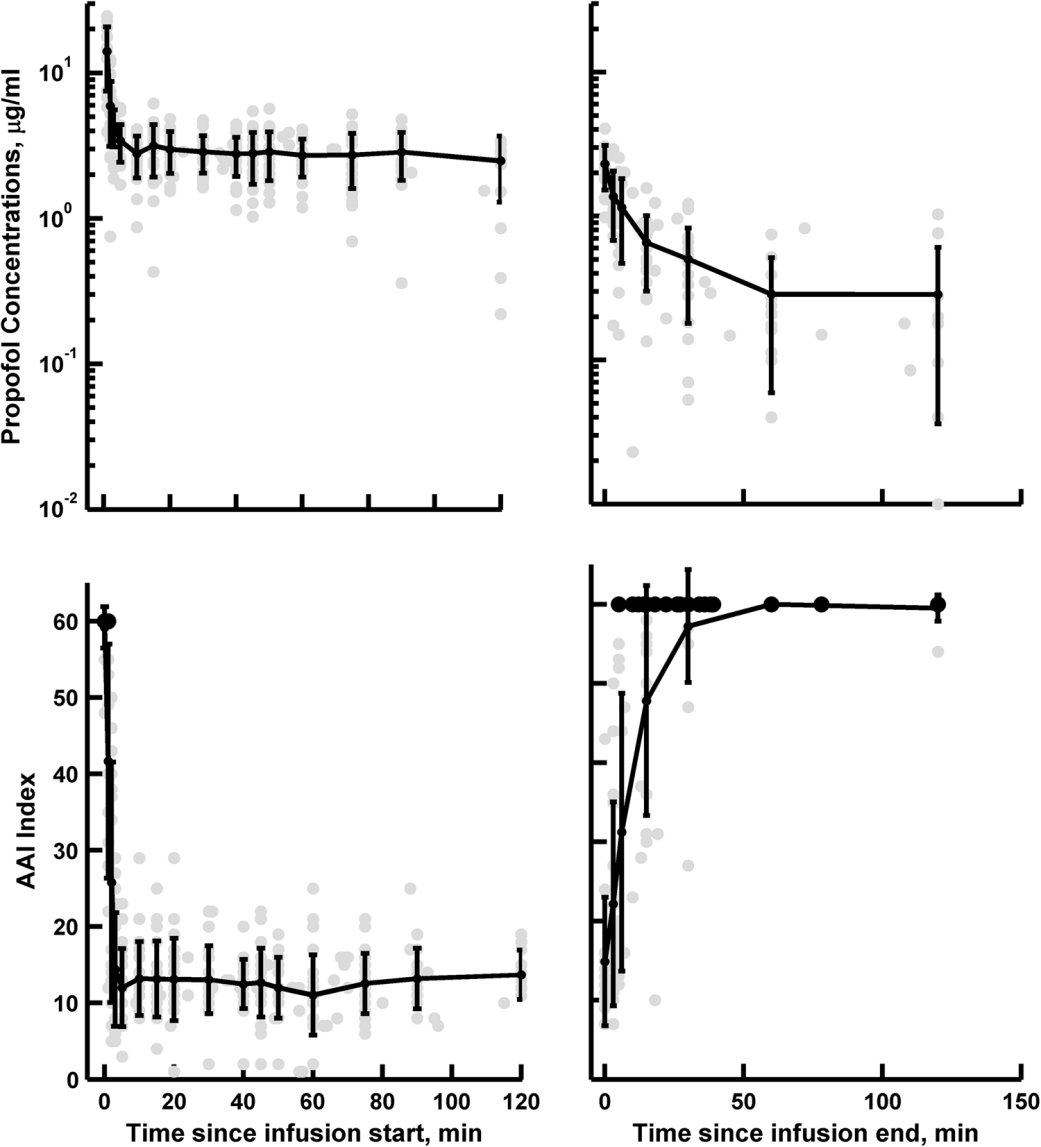
The mean ± standard deviation of propofol concentrations and AAI responses observed during the major lung surgery. The *black dots* denotes AAI index values above 60

This analysis used the concentration–time profiles and AAI measurements of propofol recorded in 23 patients scheduled for a major lung surgery. Figure 1 shows the available experimental data. It contained 423 propofol concentrations and 462 AAI index measurements. Table 1 lists the summary of patients’ demographic data. The model-building process started with a three-compartment model [16, 26–29], which turned out to be sufficient to describe our data. The use of a simpler two-compartment model was not superior as indicated by the ΔMOF = 25.7 (p < 0.001). The sigmoidal *E*_*max*_ model was used for the pharmacodynamic data. The AAI index was directly related with the concentration of propofol in the biophase (effect) compartment. The use of a two-compartment effect site model, as proposed by Björnsson et al. [27], did not improve model predictions.

**Table 1:** Demographic characterization of patients included in the study

Parameter (unit)	Median [range] n = 23
Age (years)	60 [51–75]
Weight (kg)	77 [44–125]
Height (cm)	172 [152–183]
Lean body mass (kg)	56.4 [34.7–77.1]
Male/female	15/8
Propofol’s infusion duration (min)	140 [67–214]
Average systolic blood pressure (mmHg)	111 [50–210]
Average diastolic blood pressure (mmHg)	72 [33–120]
Average heart rate (beats/min)	71 [48–114]
Baseline systolic blood pressure (mmHg)	128 [92–200]
Baseline diastolic blood pressure (mmHg)	77 [59–110]
Baseline heart rate (beats/min)	70 [52–92]

Results are expressed as median and range

**Fig. 2:**
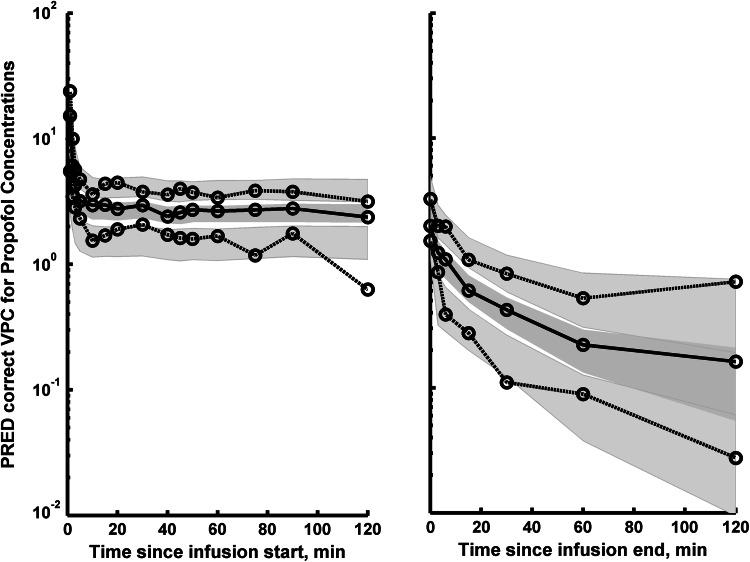
The prediction corrected visual predictive check (pcVPC) for propofol concentrations. The VPC plots show the simulation-based 95 % confidence intervals around the 10th, 50th, and 90th percentiles of the PK data in the form of *blue* (50th) and *gray* (10th and 90th) areas. The corresponding percentiles from the prediction corrected observed data are plotted in *black color* (Color figure online)

**Fig. 3:**
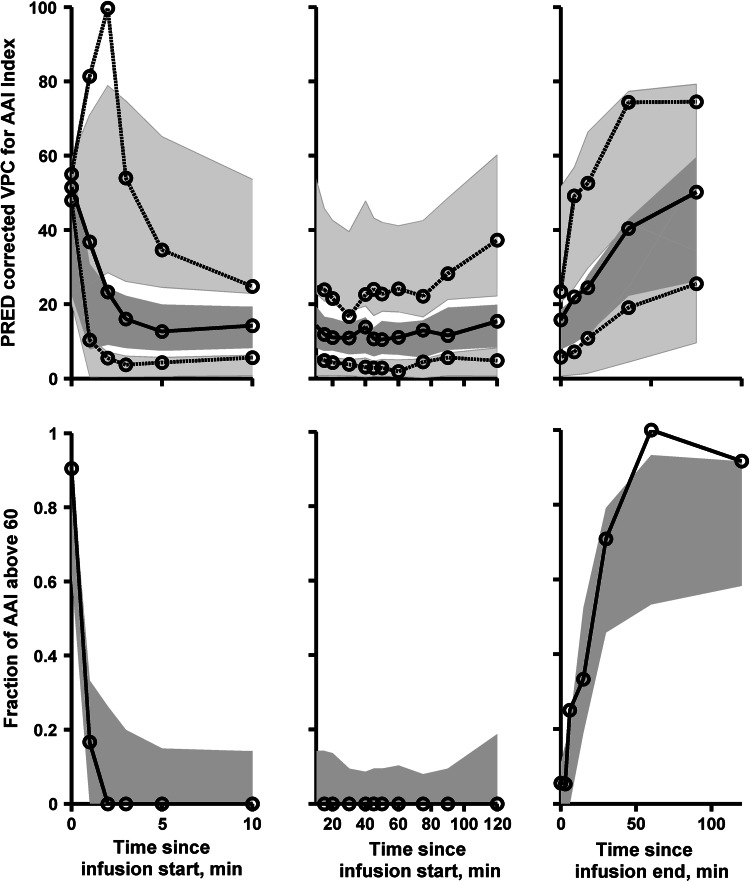
The prediction corrected visual predictive check (pcVPC) for AAI index. The *upper panels* show the simulation-based 95 % confidence intervals around the 10th, 50th, and 90th percentiles of the PD data in the form of *blue* (50th) and *gray* (10th and 90th) areas. The corresponding percentiles from the prediction corrected observed data are plotted in *black color*. The *lower panels* show simulation based 95 % confidence intervals (*blue* are) for the fraction of AAI observations above 60. The observed fraction of AAI observations above 60 are represented with a *black color* (Color figure online)

The typical goodness-of-fit plots of the final PK/PD model are provided in the Supplementary materials. The individual predictions are very close to the experimental data, indicating good performance of the model, which is also confirmed by other goodness-of-fit plots. The pcVPC for the propofol concentration and AAI were used to assess the simulation properties of the model. Figure 2 shows the results for PK and Fig. 3 for PD. pcVPC plots indicate that both the central tendency of the data and the variability at a particular sampling time were recaptured well. There are no misspecifications for the PK part of the model and some small deviations in the 10th and 90th percentile for the AAI index as a slightly higher variability is predicted by the model than is supported by the data. Nevertheless, for this relatively small dataset the overall prediction capabilities of the model are acceptable.

Tables 2 and 3 provides the final parameter estimates along with bootstrap results. In a 1,000-run bootstrap analysis 13 (1.3 %) for PK and 8 (0.8 %) for PD runs terminated due to rounding errors and were excluded from the analysis. All PK/PD parameters, inter-subject and residual error variances were estimated with low (lower than 50 %) coefficients of variation. There are no major difference between bootstrap and NONMEM derived standard error of estimates. The shrinkage was acceptable except for the *V*_*T1*_, *Q*_2_, and *V*_*T2*_, for which it was 100 % (the data were not informative with regard to the inter-patient variability of those parameters).

**Table 2:** The parameter estimates of the final PK model of propofol

Parameter (unit)	Description	θ, Estimate (% CV) [5th–95th CI]	ω^2^, Estimate (%CV) [shrinkage] [5^th^–95th CI]	θ, Bootstrap median [5th–95th CI]	ω^2^, Bootstrap median [5th–95th CI]
*V*_*P*_ (L)	Volume of central compartment	5.11 (17.9) [3.61–6.61]	73.3 (31.8) [9.3] [35.0 – 112]	5.01 [3.38–8.98]	71.6 [0.3–104]
*CL* (L/min)	Clearance	2.38 (8.4) [2.07–2.71]	21.7 (42.6) [3.6] [6.5–36.9]	2.38 [1.98–2.72]	21.0 [14.2–28.9]
*V*_*T1*_ (L)	Volume of distribution of the peripheral compartment	14.2 (33.2) [6.44–21.9]	0 FIX^a^ [100]	14.2 [7.32–21.3]	–
*Q*_*1*_ (L/min)	Distribution clearance	1.17 (14.5) [0.891–1.45]	0 FIX^a^ [100]	1.15 [0.856–1.52]	–
*V*_*T2*_ (L)	Volume of distribution of the peripheral compartment	189 (44.6) [50.3– 327]	0 FIX^a^ [100]	178 [100–458]	–
*Q*_*2*_ (L/min)	Distribution clearance	0.608 (46.3) [0.145–1.07]	59.3 (46.3) [9.65] [14.4–104]	0.584 [0.258–0.934]	60.6 [39.2–118]
Residual error model					
$\sigma_{{{\text{prop}},{\text{ Cp}}}}^{2}$	Proportional residual error variability	30.0 (6.3) [5.6] [26.9–33.1]		29.6 [26.4–33.1]	

The bootstrap estimates are given for comparison

^a^Fixed as they tended to zero or were insignificant during the model building process

**Table 3:** The parameter estimates of the final PK model of propofol

Parameter (unit)	Description	θ, Estimate (% CV) [5th–95th CI]	ω^2^, Estimate (%CV) [shrinkage] [5th–95th CI]	θ, Bootstrap median [5th–95th CI]	ω^2^, Bootstrap median [5th–95th CI]
*AAI*_*0*_	Baseline AAI index	87 (fixed)^a^	–	87 (fixed)	–
*E*_*MAX*_	Maximal effect	1 (fixed)	–	1 (fixed)	–
*Ce*_*50*_ (mg/L)	Effect site concentration needed to reach 50 % of *E*_*max*_	1.40 (9.3) [1.18–1.61]	25.6 (19.1) [9.2] [17.6–33.6]	1.37 [1.15–1.59]	23.2 [14.6–31.4]
γ	Shape factor	2.76 (14.3) [2.11–3.41]	39.9 (17.5) [6.4] [28.4–51.4]	2.66 [2.02–3.36]	37.3 [3.45–48.8]
*k*_*e0*_ (1/min)	Rate constant for distribution from effect compartment	0.103 (10.7) [0.085–0.121]	43.4 (15.3) [5.1] [32.5–54.3]	0.103 [0.087–0.125]	42.8 [32.7–55.2]
Residual error model					
$\sigma_{{{\text{add}},{\text{ AAI}}}}^{2}$	Additive residual error variability	0.553 (48.8) [5.2] [0.11–1.00]		0.616 [0.005–0.891]	
$\sigma_{{{\text{prop}},{\text{ AAI}}}}^{2}$	Proportional residual error variability	31.8 (6.7) [5.2] [28.3–35.3]		32.0 [28.8–35.5]	

The bootstrap estimates are given for comparison

^a^Fixed based on study [41]

**Fig. 4:**
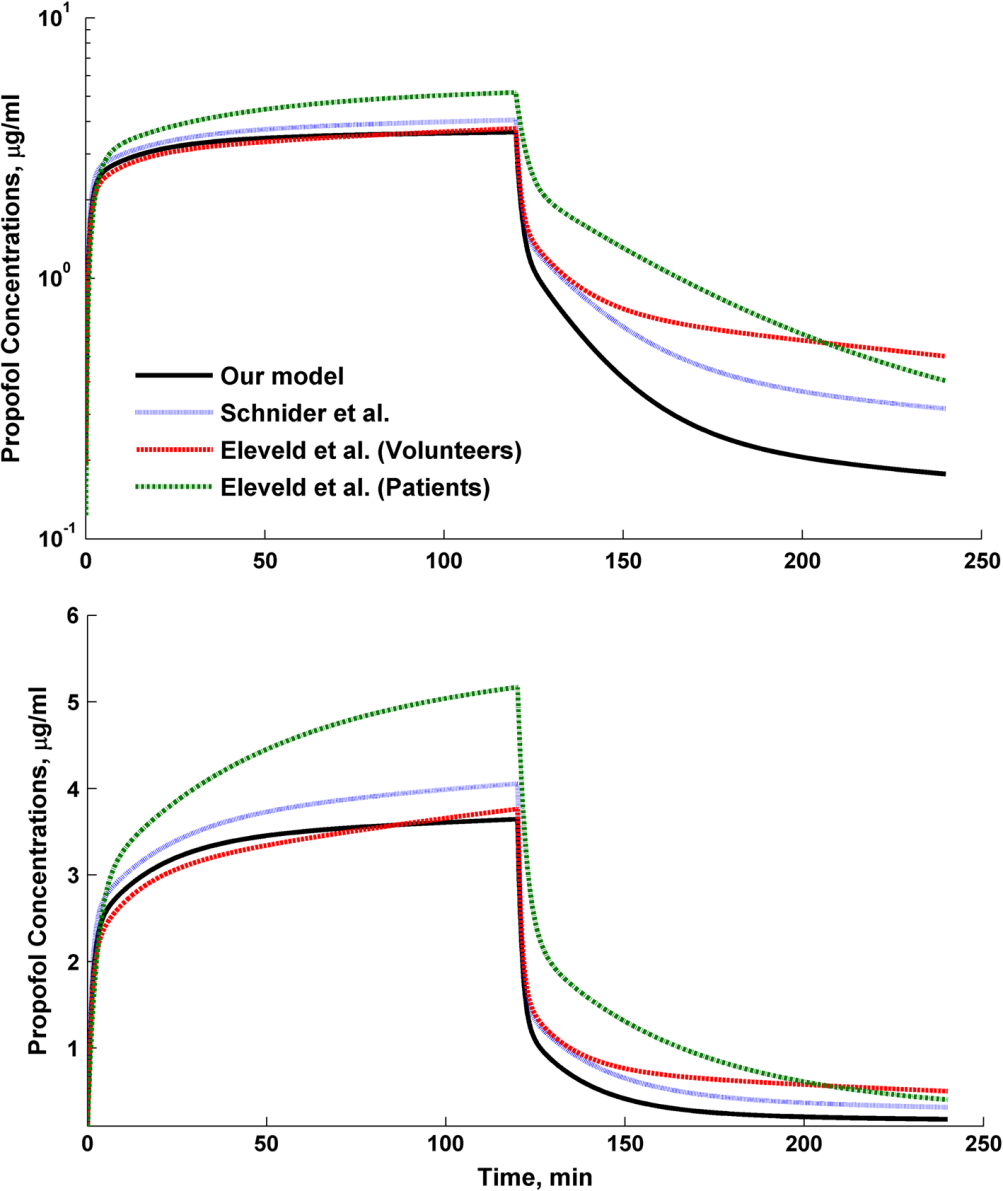
The comparison of Schnider et al. [15], Eleveld et al. [16] (patients and volunteers) and this study model assuming typical parameter estimates adjusted to the typical patient of this study. The infusion duration of 120 min and infusion rate of 8 mg/kg/h were used for simulations. The linear and logarithmic scale was applied to *Y* axis

The typical value of the volume of the central compartment (*V*_*T1*_) was 5.11 L, whereas the volumes of the peripheral compartments (*V*_*T2*_ and *V*_*T3*_) were 14.2 L and 189 L, respectively. The systemic clearance (*Cl*) of propofol was 2.38 L/min (0.0333 L/min/kg). The clearances between the central and both peripheral compartments (*Q*_*2*_ and *Q*_*3*_) were 1.17 and 0.608 L/min, respectively. The IIV was only estimated for the *V*_*C*_, *CL* and *Q*_*3*_, for which it amounted to 73, 22 and 59 %. For the other PK parameters it tended to zero or was insignificant during the model building process. The maximum effect of propofol, which produces the deepest level of anesthesia, was fixed to one. The concentration of propofol in the plasma that produces 50 % of the maximum effect (*Ce*_*50*_) was 1.40 mg/L, with modest variability of 26 %. The gamma was high (2.76) and variable (40 %), indicating a steep relationship between the AAI and biophase concentrations of propofol. The high gamma suggest that any changes in the biophase concentrations of propofol lead to the large changes in the AAI index. The biophase distribution rate constant was 0.103 min^−1^ with high variability of 43 %. It corresponds to the half-life of 6.72 min for a typical patient (Fig. 4).

**Fig. 5:**
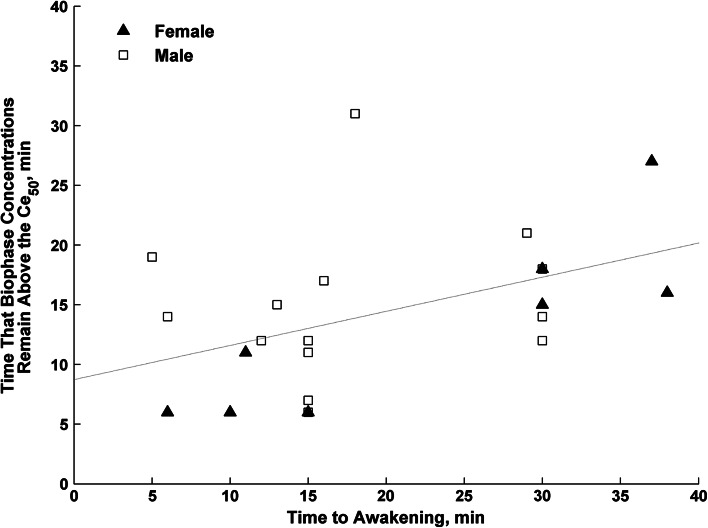
Relationship between time that biophase concentrations remain above the *Ce*_*50*_ and experimentally observed time to awakening. The *broken line* is a regression line (R^2^ = 0.200, p = 0.034)

The median time to awakening after cessation of propofol infusion was 15 min and ranged between 5 and 38 min. The propofol concentrations in plasma and biophase and AAI index value were determined from the individual prediction of the final model. The median (range) propofol concentration in the plasma was 0.60 (0.20–1.96), the biophase concentration of propofol was 1.13 mg/L (0.48–3.08 mg/L) and the AAI index was 55.1 (21.3–82). A highly skewed distribution of the AAI values at the time of orientation was noted, where most patients’ (80 %) AAI values ranged from 50 to 60. Figure 5 shows the relationship between time to awakening and time that biophase concentrations remain above the *C*_*e50*_. The lower associations were observed for *C*_*e20*_ and *C*_*e10*_.

The covariate search comprised the assessment of various demographic and clinical parameters like body weight, gender, age, blood pressure, heart rate, laboratory blood tests results, and stage of lung cancer on the individual PK/PD parameter estimates. No statistically significant relationships (p < 0.01) were identified in this study.

Table 4 compares the Schnider et el. [15], Eleveld et al. [16] (patients and healthy volunteers), and this study fixed effect estimates and models performance as reflected by bias (MDPE) and accuracy (MDAPE). The best predictions were obtained for Eleveld model assuming PK parameters for healthy volunteers and Schnider model. Surprisingly, the worst predictions were obtained for Eleveld model assuming PK parameter for patients. It suggests that propofol PK for lung cancer patients undergoing major lung surgery is similar to that observed in healthy subjects. The major difference (larger than 20 %) between the Eleveld volunteers and our model were noted for Q_2_ (−136 %) and *V*_*T2*_ (−30 %) and *V*_*T1*_ (26 %), whereas between the Schnider and our model for *Q*_*2*_ (−38 %), CL (28 %), and *V*_*T2*_ (−26 %). These disparities translate to small differences in PK profiles as illustrated in Fig. 4. As can be expected, the considerable over-predictions are present for the Eleveld model for patients.

**Table 4:** The comparison of typical estimates obtained in this study (surgery cancer patients) and results obtained by Schnider et al. (volunteers) and Eleveld et al. (patients and volunteers)

Parameter (unit)	This study, patients	Schnider, volunteers		Eleveld, volunteers		Eleveld, patients	
	Typical (median)	Typical (median)	Bias (%)	Typical (median)	Bias (%)	Typical (median)	Bias (%)
*V*_*P*_ (L)	5.11	4.27	16	5.18	−1.4	8.16	−60
*CL* (L/min)	2.38	1.94	28	1.96	18	1.64	31
*V*_*T1*_ (L)	14.2	16.2	−14	10.5	26	31.4	−121
*Q*_*1*_ (L/min)	1.17	1.12	4.3	1.20	−4.6	1.27	−8.5
*V*_*T2*_ (L)	189	238	−26	231	−22	105	44
*Q*_*2*_ (L/min)	0.608	0.836	−38	1.38	−127	0.41	32
*MDPE*	−0.50	−8.4		−1.0 %		−29.4	
*MDAPE*	12.1	13.6		9.3 %		29.4	

All parameters were scaled to the typical patient of this study. The bias indicates the relative difference in parameters between studies. The median prediction error (MDPE) and median absolute prediction error (MDAPE) are provided to compare models performance

## Discussion

The main aim of this study was to investigate the PK/PD of propofol in cancer patients, because the information available in the literature concerning this ever-growing population is still sparse. We also assessed whether routinely recorded covariates could explain the inter-patient variability observed in the PK/PD of propofol. Finally, we compared our data with the Schnider et al. model, which is currently incorporated in commercial target-controlled infusion pumps for the administration of propofol, and with the Eleveld et al. model, which is the most comprehensive model of propofol available in the literature.

The PK parameter estimates of the present model were similar to those obtained (and scaled to the typical values of this study) by Schnider et al. [15] and Eleveld et al. [16] for healthy volunteers. The highest difference was observed for distribution clearance and volume of distribution associated with the “deep” compartment. The clearance estimate obtained in this study was about 20 % higher than one estimated by Schnider and Eleveld for healthy volunteers, and about 30 % higher than one estimated by Eleveld for patients. To some degree these dissimilarities may arise from differences in the study designs. In the Schnider’s study [15] the volunteers received propofol only for the purpose of the study, without any concomitant drugs or surgical procedures. Thoracic surgery with sympathicolysis due to epidural anesthesia, unilateral positioning and one-lung ventilation, as was the case with the patients in the present study, could certainly affect the pharmacokinetics. Hypoxia, oxidative stress and changes in the cardiac output occurring during this kind of surgery certainly influence what happens to the drug in the organism at least as well as a different sampling schedule. An increase in the volume of peripheral compartment with prolonged infusion of propofol is also a well-known phenomenon associated with the high lipophilicity of propofol [32]. Nevertheless, these differences do not translate into significant differences in propofol concentrations during the infusion. The clearance of propofol observed in this study was also slightly larger than in other studies available in the literature including both healthy individuals as well as ASA I–III patients scheduled for laparoscopic cholecystectomy [2, 8, 11, 27, 29–32]. Occasionally, even higher clearances have been observed for ASA III patients undergoing aortic surgery (2.64 L/min) [33]. This suggests that PK of propofol has not been fully understood in all clinical situations, and further research is necessary to indentify all the mechanisms that underlie the observed differences.

Patients operated due to lung cancer are a special population. They may demonstrate different abnormalities in laboratory data and organ function. The following co-morbidities have been diagnosed in the population under study: hypertension, diabetes, major depression, obesity, chronic obstructive pulmonary disease, renal failure, gastritis, hyperthyroidism, atrial fibrillation, coronary artery disease and post-myocardial infarction status. Besides, in some patients hypoalbuminemia and an increased level of leukocytes was observed. Despite the collection of a large number of covariates, none of them significantly affected the PK/PD of propofol. Interestingly, the clearance was found to be independent of the body weight despite the significant variability of this parameter in our group (44–125 kg). This might have clinical implications, potentially resulting in a simplification and standardization of propofol dosage, although a larger group of patients needs to be studied to validate this statement.

The modeling of the AAI index led to the estimation of basic parameters reflecting the pharmacodynamics of propofol. The *Ce*_*50*_ (1.40 mg/L) was significantly lower than the BIS-derived *Ce*_*50*_ in ASA I–II surgical patients [34, 35] and ASA III patients undergoing an aortic surgery [33]. It may indicate that lung cancer patients are more sensitive to propofol anesthesia. However, the rather low value of the *Ce*_*50*_ observed in this study cannot be simply recognized as a higher sensitivity to propofol anesthesia. Above all, it is difficult to compare different monitors and measures of the depth of anesthesia used in the literature. Most of the literature data concerning the pharmacodynamics of propofol were obtained from studies with the BIS monitor. The other factors, which might have contributed to the increased sensitivity to propofol and which limit the interpretation of the obtained low value of *Ce*_*50*_, are the patients’ age and health status, as well as the premedication with benzodiazepines and co-administration of fentanyl. We gave our patients fentanyl, which has strong analgesic potency and due to some hypnotic activity, it may affect the EEG signal. As far as the full recovery with the orientation for name and place is concern, the recovery times obtained in our study were similar to the literature values for ASA I–II patients undergoing TIVA [8, 36, 37], whereas the median value of propofol concentration in the biophase at the orientation (1.13 mg/L) was lower when compared with the literature data. There is moderate, but statistically significant, relationship between patients’ time to awakening and the time the propofol biophase concentrations remain above the *Ce50,* suggesting the usefulness of AAI index in predicting time to patients orientation after infusion cessation. The lack of stronger association might be a consequence of the opioid use that is a known factor influencing recovery parameters [39]. Mi et al. noted [38] that the concentrations of propofol at the recovery depend on the actual fentanyl concentrations in the plasma. Our values of propofol concentrations at the recovery were even lower than the ones obtained by Mi et al. (2.1 mg/L) for high fentanyl concentrations. So taking the data obtained by Mi et al. into account, where the same opioid was used, our recovery time point concentrations may indicate lung cancer patients’ higher sensitivity to propofol anesthesia.

The sensitivity of cancer patients to propofol has been poorly investigated so far. The only study addressing this problem is the one by Chan et al. [40], who reported a very low value of the median effective dose of propofol in patients with a brain tumor. It is noteworthy that according to the results obtained by Chan et al., only large tumors significantly increased the potency of propofol. On the other hand, one of the factors which cannot be excluded as a cause of such differences may be the neurotoxicity of cancer chemotherapy, which is a common and potential dose-limiting complication of cancer chemotherapy.

To conclude, we identified only small differences in the pharmacokinetics of propofol in cancer patients, as compared with the Schnider and Eleveld model for healthy volunteers. Nevertheless, the low bias of predictions does not necessitate the modification of propofol dosage in the population under study when TCI-guided administration of propofol by means of the Schnider model is used. This is of clinical importance, because the propofol TIVA is increasingly used for cancer surgery, so commercially used TCI models may be used in this population. The Eleveld model with parameters for healthy volunteers also leads to an excellent predictions and can be used to guide TCI pumps. However, its counterpart with PK parameters for patients considerably over-predicts propofol concentrations and should be used with caution. The pharmacodynamics of propofol remains an open question. However, cancer patients’ increased sensitivity to propofol cannot be excluded.

Supplementary material is available and includes the clinical characteristic of patients, goodness-of-fit plots, and correlation plots for individual PK/PD parameters versus possible covariates body weight, and age.

## Electronic supplementary material

Below is the link to the electronic supplementary material.

Supplementary material 1 (DOCX 1195 kb)
