# Supplementary material for: Pharmacokinetics and pharmacodynamics of propofol in cancer patients undergoing major lung surgery
Source: J Pharmacokinet Pharmacodyn. 2015 Jan 28;42(2):111–22. doi: 10.1007/s10928-015-9404-6 (PMC4355445; doi:10.1007/s10928-015-9404-6)
Supplement: Supplementary file 1 — Supplementary material 1 (DOCX 1195 kb) [file 10928_2015_9404_MOESM1_ESM.docx]

**Supporting Online Material to “Pharmacokinetics and pharmacodynamics of propofol in cancer patients undergoing major lung surgery”**

Krzysztof Przybyłowski, Joanna Tyczka, Damian Szczesny, Agnieszka Bienert, Paweł Wiczling, Katarzyna Kut_,_ Emilia Plenzler, Roman Kaliszan, Edmund Grześkowiak

Email: wiczling@gumed.edu.pl

Table 1S. Clinical characteristic of patients enrolled into the study. These parameters were included in the covariate analysis.

| **Parameter, unit** | **Median [Range] n=23** |
| --- | --- |
| **Laboratory blood tests results:**  RBC, M/µl  Hb, g/dl  HCT, %  WBC, K/µl  Lymphocytes, K/µl  Lymphocytes, %  Neutrophils, K/µl  Neutrophils, %  Platelets, K/µl  APTT, s  Na, mmol/l  K, mmol/l  ALT, U/L  AST, U/L  Bilirubin, mg/dl  Creatinine, mg/dl  Urea, mg/dl  Total Protein, g/dl  Glucose, mg/dl  Cholesterol, mg/dl  pH  Albumin, g/dl  Triglycerides, g/dl | 4.53 [3.58-5.69]  13.6 [9.7-16.8]  41.3 [30.5-49.7]  7.8 [4.8-37.4]  2.1 [1-3.6]  24.4 [8.8-32.7]  5.0 [3.2-32.4]  65 [57.7-86.6]  236 [169-700]  31.1 [24.6-37.4]  139 [130-144]  4.2 [3.9-5]  18 [4-92]  16 [6-27]  0.4 [0.2-1.4]  0.67 [0.48-1.17]  31 [18-61]  6.8 [6-7.3]  104 [81-211]  222 [124-286]  7.42 [7.39-7.45]  3.9 [2.4-4.5]  127 [82-272] |
| **Stage of lung cancer:**  IA/IB/IIA/IIB/IIIA | 6/2/6/4/4 |
| **Type of surgery:**  Lobectomy  Pneumonectomy  Sublobar Resection  Mediastinoscopy Exploratory Thoracotomy | 17  3  1  1  1 |
| **Comorbidities:**  Hypertension Diabetes  Obesity Chronic Obstructive Pulmonary Disease Renal Failure Gastritis  Major Depression  Hyperthyroidism  Atrial Fibrillation Coronary Artery Disease Post-Myocardial Infarction Status | 10 3 1 1 1 1 2 1 1 1 2 |


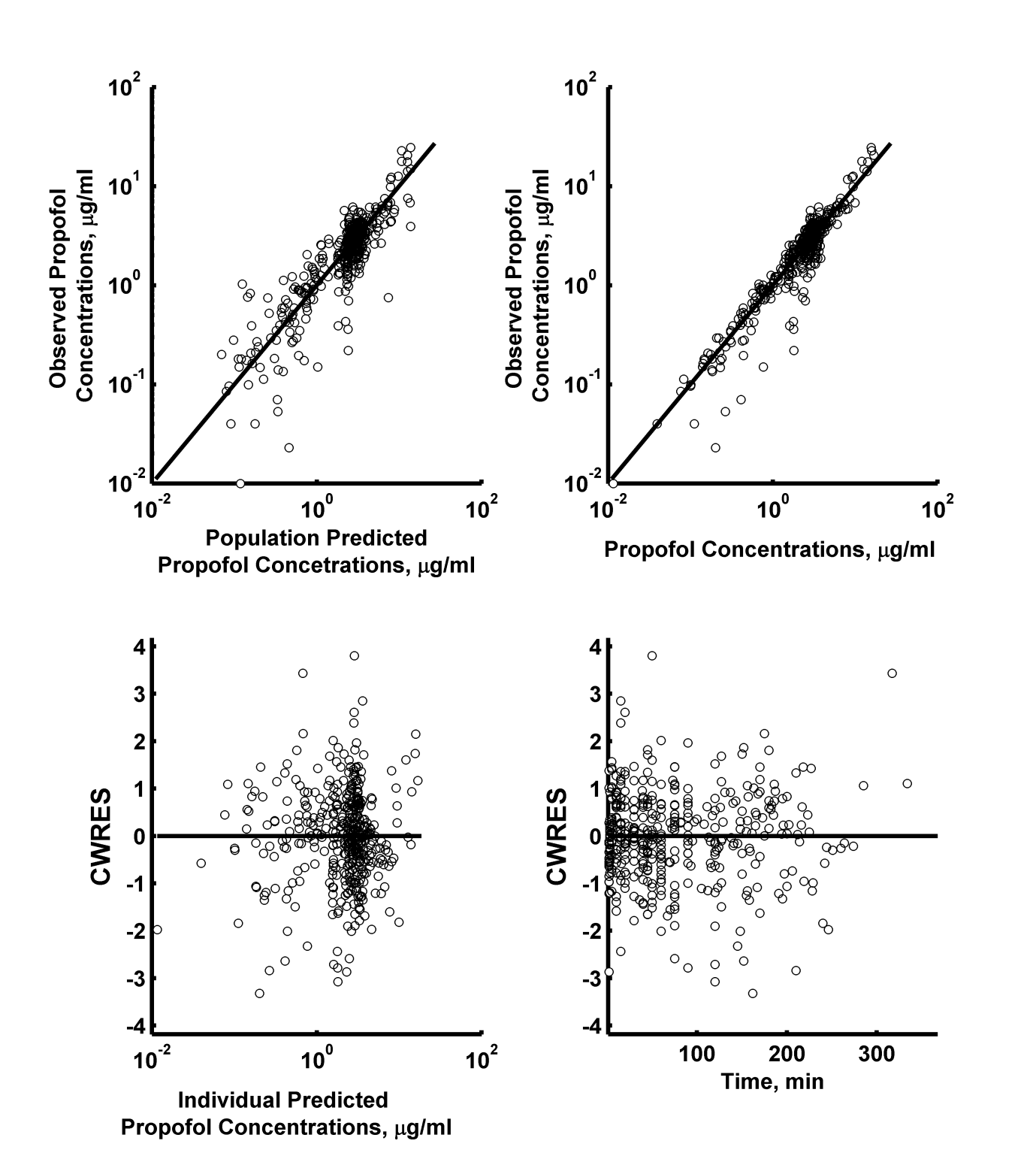


Figure 1S. Goodness of fit plots for propofol concentrations: the observed *versus* the population predicted concentrations; the observed *versus* the individual population predicted concentrations; conditional weighted residuals (CWRES) *versus* individual predicted concentrations and time.


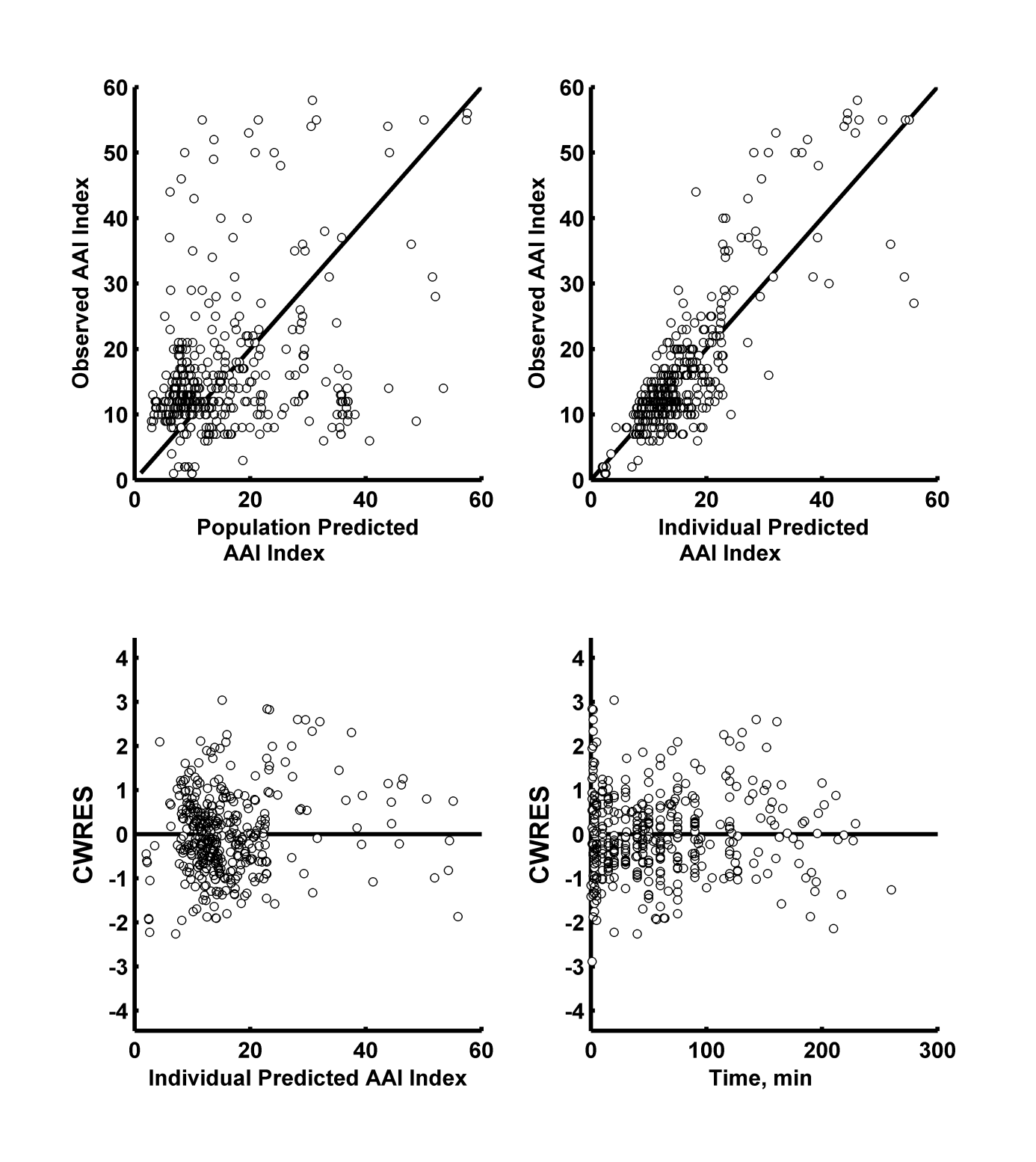


Figure 2S. Goodness of fit plots for the pharmacodynamic response (AAI Index): the observed *versus* the population predicted concentrations; the observed *versus* the individual population predicted concentrations; conditional weighted residuals (CWRES) *versus* individual predicted concentrations and time.


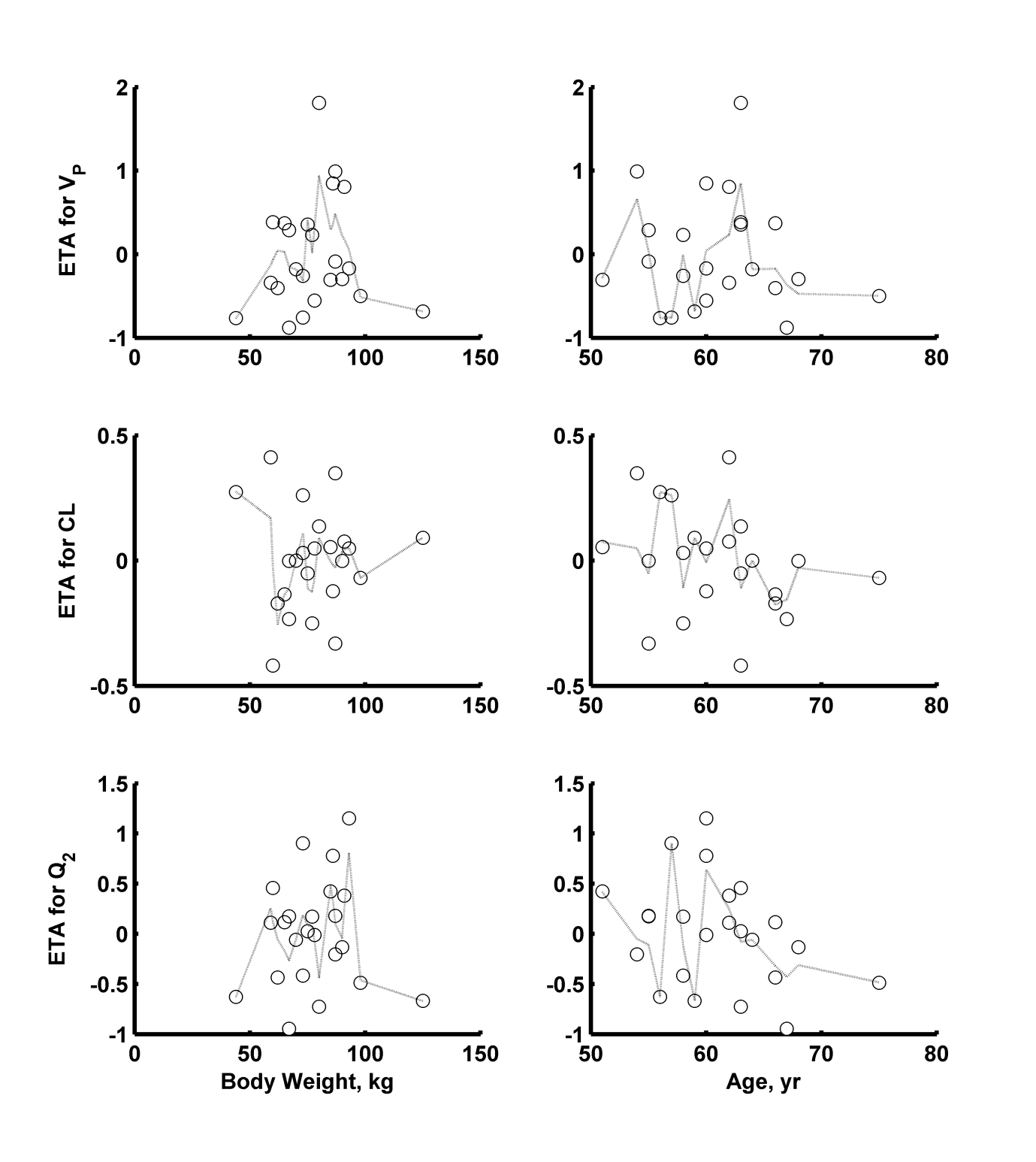


Figure 3S. The individual estimates for eta (deviation of the individual estimate from the population mean) of propofol *V_P_*_,_ *CL* and *Q_2_* in relation to the individual values of body weight and age. The dotted line indicates the trend in the data (loess smooth).


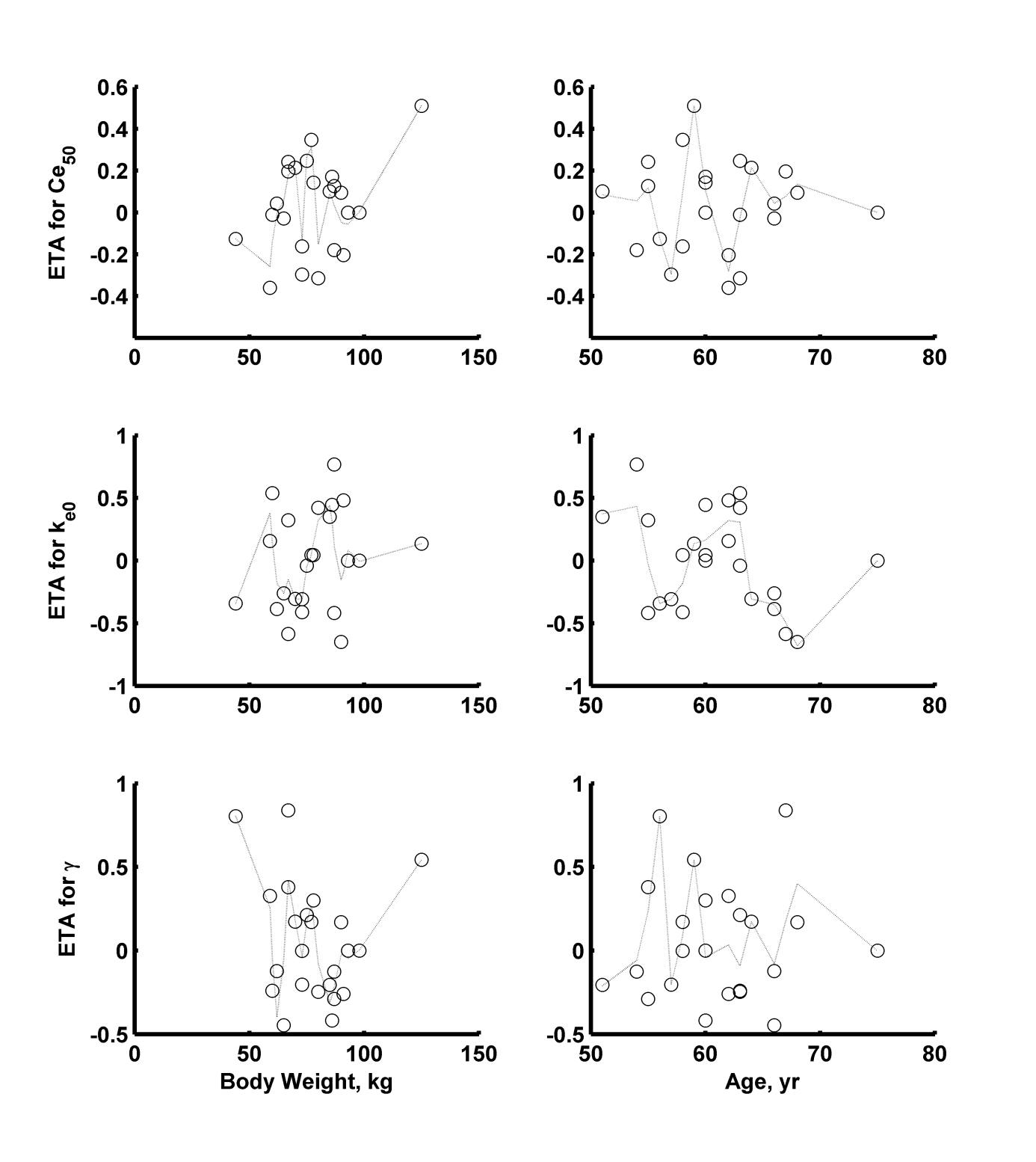


Figure 4S. The individual estimates for eta (deviation of the individual estimate from the population mean) of propofol *Ce_50_*_,_ *k_e0_* and *γ* in relation to the individual values of body weight and age. The dotted line indicates the trend in the data (loess smooth).
